# Risk Factors for Poor Outcomes in Children Hospitalized With Virus-associated Acute Lower Respiratory Infections: A Systematic Review and Meta-analysis

**DOI:** 10.1097/INF.0000000000004258

**Published:** 2024-01-26

**Authors:** Daira Trusinska, Si Thu Zin, Emmanuel Sandoval, Nusrat Homaira, Ting Shi

**Affiliations:** * From the Usher Institute, Old Medical School, Teviot Place, University of Edinburgh, Edinburgh, United Kingdom; † Randwick Clinical Campus, School of Clinical Medicine, UNSW Sydney, Sydney, New South Wales, Australia; ‡ Department of Acute and General Medicine, Royal Infirmary of Edinburgh, Edinburgh, United Kingdom; § Discipline of Pediatrics and Child Health, School of Clinical Medicine, UNSW Sydney, Sydney, New South Wales, Australia; ¶ Respiratory Department, Sydney Children’s Hospital, Randwick, Sydney, New South Wales, Australia; ∥ James P. Grant School of Public Health, BRAC University, Bangladesh.

**Keywords:** risk factor, poor outcome, respiratory syncytial virus, SARS-CoV-2, influenza

## Abstract

**Background:**

Acute lower respiratory infection (ALRI) caused by respiratory viruses is among the most common causes of hospitalization and mortality in children. We aimed to identify risk factors for poor outcomes in children <5 years old hospitalized with ALRI caused by respiratory syncytial virus (RSV), influenza and severe acute respiratory syndrome coronavirus 2 (SARS-CoV-2).

**Methods:**

We searched Embase, Medline and Global Health databases and included observational studies reporting risk factors for poor outcomes (defined as use of supplemental oxygen, mechanical ventilation, intensive care unit admission, prolonged hospital stay and mortality) published between January 2011 and January 2023. Two authors independently extracted data on study characteristics, outcomes and risk factors. Due to limited data, meta-analyses were only conducted for RSV-ALRI poor outcome risk factors using random effects model when there were at least 3 studies.

**Results:**

We included 30 studies. For RSV-related ALRI, significant risk factors based on meta-analysis were: neurological disease [odds ratio (OR): 6.14; 95% confidence intervals (CIs): 2.39–15.77], Down’s syndrome (5.43; 3.02–9.76), chronic lung disease (3.64; 1.31–10.09), immunocompromised status (3.41; 1.85–6.29), prematurity (2.98; 1.93–4.59), congenital heart disease (2.80; 1.84–4.24), underlying disease (2.45; 1.94–3.09), age <2 months (2.29; 1.78–2.94), age <6 months (2.08; 1.81–2.39), viral coinfection (2.01; 1.27–3.19), low birth weight (1.88; 1.19–2.95) and being underweight (1.80; 1.38–2.35). For influenza-related ALRI, chronic conditions and age 6–24 months were identified as risk factors for poor outcomes. Cardiovascular disease, immunosuppression, chronic kidney disease, diabetes and high blood pressure were reported as risk factors for mortality due to SARS-CoV-2 associated ALRI.

**Conclusions:**

These findings might contribute to the development of guidelines for prophylaxis and management of ALRI caused by RSV, influenza and SARS-CoV-2.

Acute lower respiratory infection (ALRI), including bronchiolitis and pneumonia, constitutes a considerable disease burden in young children around the world. Bronchiolitis is common in children under 2 years of age, whereas pneumonia is seen in people of all ages.^1,2^ Although between 1990 and 2017 the global incidence of ALRI in children under 5 years of age decreased by 32.4%, in 2017 ALRI was found to have caused 808,920 deaths in this age group.^3^ The pneumonia etiology research for child health study has shown leading pathogens responsible for severe pneumonia cases requiring hospital admission in children younger than 5 years across a wide range of geographical and epidemiological settings, including respiratory syncytial virus (RSV) and influenza.^4^ Moreover, the most substantial disease burden is seen in developing countries with an estimated 82% of all influenza-associated and over 97% of RSV-associated ALRI deaths.^5,6^ In recent years, the emergence of the severe acute respiratory syndrome coronavirus 2 (SARS-CoV-2) has impacted the epidemiological landscape of other respiratory viruses, highlighting the need to continuously monitor the prevalence and severity of diseases caused by respiratory viruses.^7^ Therefore, this study focuses on risk factors for poor outcomes related to severe ALRI caused by RSV, influenza and SARS-CoV-2 requiring hospitalization in children under 5 years old.

According to a systematic analysis, influenza causes 870,000 annual hospitalizations in children under 5 years old.^8^ The recently emerged SARS-CoV-2 has shown low severity in children: UNICEF estimates that children 0–4 years old only constitute 3.4% of all reported infections and 0.1% of total SARS-CoV-2 related deaths.^9^ In contrast to influenza and SARS-CoV-2, RSV disease burden is mostly experienced by young children.^6,10^ In 2019, RSV caused approximately 3.6 million hospital admissions and 26,300 ALRI in-hospital deaths in children under 5 years old, globally.^6,11^ Although RSV is not necessarily more severe, its high burden in very young children with ALRI necessitates continuous surveillance and research.

Data suggest that 14% of children hospitalized with RSV will develop respiratory failure with 8% developing apneic episodes and children with respiratory complications are significantly more likely to be hospitalized for a longer period compared to children without any complications.^12^ In extreme situations, respiratory complications such as pneumothorax, pleural effusion and sepsis may be spurred on by RSV.^12,13^ Several risk factors for poor outcomes, mostly due to RSV infection, have been identified. For instance, 1 study identified the presence of comorbidities, congenital heart disease, prematurity and young age as significant risk factors for poor outcomes.^14^ Another 2 studies have reported bronchopulmonary dysplasia and Down’s syndrome as significantly associated with severe disease.^15,16^ The mortality rate has been estimated to be 18.8 times higher in children with risk factors compared to children with no risk factors.^17^

The heterogeneity of studies that report risk factors of poor outcomes in children hospitalized with ALRI frequently leads to differing and controversial conclusions, making it challenging to draw robust conclusions. Previous studies have also noted differences in the strengths of association between risk factors and outcomes in children of different age groups and world regions, highlighting the need for a nuanced analysis of subgroups.^6^ Identification of the risk factors for more severe outcomes in our review may contribute to the development of guidelines for cost-effective and targeted prophylaxis and management of these infections, as well as reduce the economic burden on healthcare systems.

Although up until early 2023, palivizumab, a humanized monoclonal antibody (mAb) was the only available prophylaxis against severe RSV disease in high-risk infants, the therapeutic landscape of RSV for all infants will change in the coming years. There are more than 30 therapeutic and prophylactic candidates in different stages of development for prevention of RSV. RSV passive immunization for newborns (extended half-life mAb) and RSV maternal vaccination are currently being considered by many countries, including the UK’s Joint Committee on Vaccination and Immunization, which has advised that both products are suitable for a universal program to protect neonates and infants from RSV.^18^ Therefore, the primary aim of this study was to identify the risk factors for poor outcomes among children under 5 years old hospitalized with RSV, influenza or SARS-CoV-2 associated ALRI. The secondary aim was to compare differences in the associations between risk factors and poor outcomes for subgroups of children stratified by region and age group. The findings will be crucial for policy decisions around prioritizing uptake of emerging RSV prophylactics.

## MATERIALS AND METHODS

### Systematic Review

We carried out a systematic review and meta-analysis according to the preferred reporting items for systematic reviews and meta-analyses guidelines.^19^ Search strategies were developed for 3 databases: Embase, Medline and Global Health (see Table, Supplemental Digital Content 1, http://links.lww.com/INF/F402). Studies that were selected for inclusion assessed risk factors for poor outcomes of ALRI related to RSV, influenza or SARS-CoV-2 in children under the age of 5. We aimed to identify all relevant studies published in English from January 1, 2011 to January 6, 2023. Only observational studies with original data and a sample size of at least 50 children with ALRI were included. One investigator (D.T.) performed the search in the databases and the initial study screening; full-text screening and data extraction were carried out independently by 2 investigators (D.T. and S.T.Z.). Any disagreements were resolved by a third investigator (T.S.).

**FIGURE 1.**
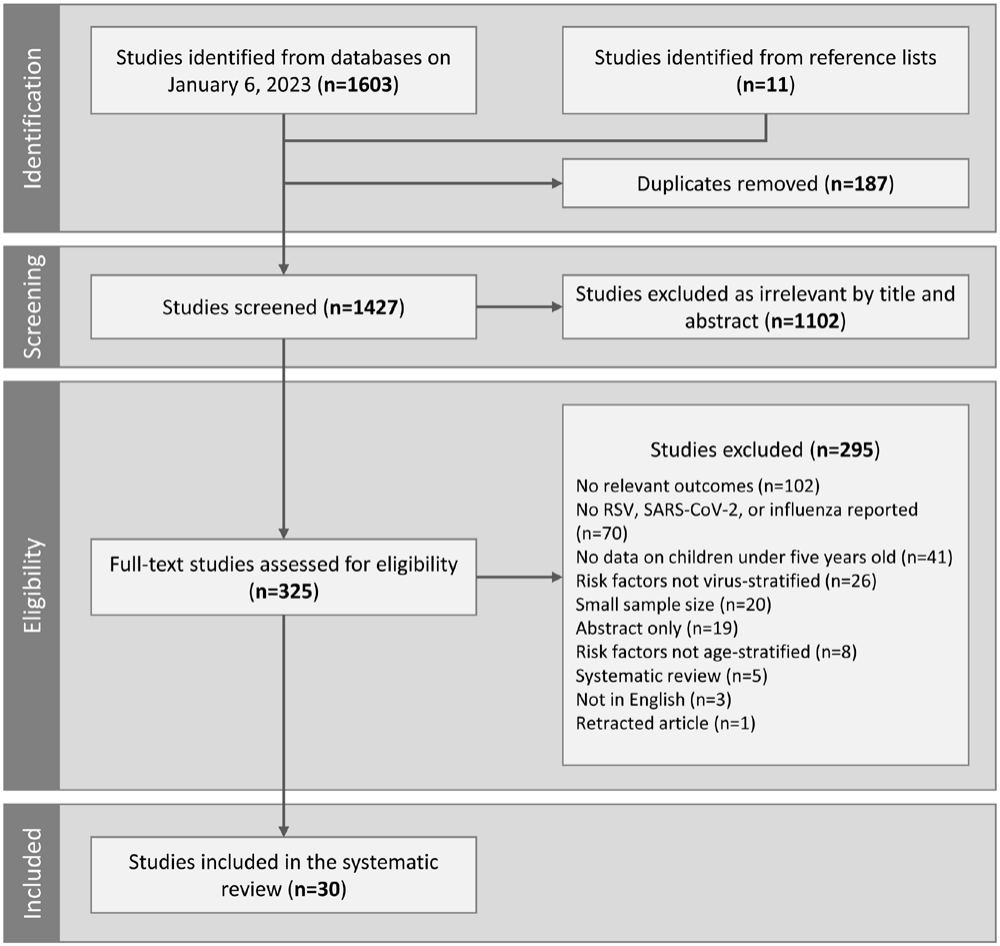
Preferred reporting items for systematic reviews and meta-analyses (PRISMA) flowchart: identification and screening of studies for inclusion in the systematic review.

The initial search identified a total of 1603 studies and another 11 studies were identified from reference lists. The screening tool Covidence was used to remove 187 duplicates. Then, the titles and abstracts of 1427 studies were screened according to selection criteria and 1102 studies were excluded as irrelevant (see Table, Supplemental Digital Content 2, http://links.lww.com/INF/F403). We screened the full texts of the remaining 325 studies. At this stage, 295 studies were excluded for several reasons such as the lack of relevant outcomes or no clearly reported RSV, influenza or SARS-CoV-2 infections (Fig. 1). Overall, 30 studies were included in the systematic review.

### Definitions

To select relevant studies, the study population was defined as children under 5 years of age hospitalized with RSV, influenza and SARS-CoV-2 associated ALRI, which included the diagnoses of bronchiolitis and pneumonia. The diagnosis of the specific pathogen was based on International Classification of Diseases codes or laboratory diagnosis. Poor outcomes were defined as the use of supplementary oxygen, mechanical ventilation, intensive care unit (ICU) transfer/admission, prolonged hospital stay (defined as more than 5 days) or mortality. The study population consisted of cases (children <5 years old hospitalized with either RSV, influenza or SARS-CoV-2 associated ALRI who developed any of the poor outcomes defined above while hospitalized) and a control group (children <5 years of age hospitalized with either RSV, influenza or SARS-CoV-2 associated ALRI who did not develop any of the poor outcomes defined above while hospitalized). The definitions for risk factors varied considerably across studies (summarized in Table, Supplemental Digital Content 3, http://links.lww.com/INF/F404).

For subgroup analyses, countries, where the included studies were carried out, were classified as either developed or developing, according to the United Nations (UN) criteria.^20^ Additionally, a subgroup meta-analysis from studies with children under 2 years of age was compared to meta-analysis from all studies.

### Quality Assessment

The quality of all the included studies was assessed using a variation of Grading of Recommendations, Assessment, Development and Evaluations scoring system.^14,21^ The included criteria were study design, control group, sample size, analysis type, avoiding bias, adjustment for confounding factors and geographical spread.

### Data Analysis

The reported odds ratios (ORs) and risk ratios (RRs), and their corresponding 95% confidence intervals (CIs) were extracted from all the included studies for each of the reported risk factors of poor outcomes. ORs or RRs represented the odds or risk that the poor outcome occurs in children hospitalized with pathogen specific (RSV, influenza or SARS-CoV-2) ALRI given a particular exposure (risk factor) compared to the odds or risk of the poor outcome occurring in the absence of that exposure. Data from univariable and multivariable studies were included and analyzed for each virus separately. Additionally, for some studies, univariable data were self-calculated if raw data were available. All meta-analyses were performed using statistical software R version 4.2.3 for risk factors reported in at least 3 studies. The heterogeneity of the studies was assessed with *I*^2^ statistic, and the random effects model was used in the meta-analysis because substantial heterogeneity across the included studies was expected. Descriptive analysis was used to summarize the risk factors of poor outcomes reported in less than 3 studies.

Subgroup analyses were conducted to compare associations between the risk factors identified in the meta-analysis and poor outcomes in developed and developing countries, as well as in study populations under 2 years of age and under 5 years of age. We carried out sensitivity analysis focusing on studies with quality assessment score of 7 and above (quality assessment scores are available in Table, Supplemental Digital Content 4, http://links.lww.com/INF/F405). Additionally, due to the broad definition used for “poor outcomes” in this review, we carried out 3 additional sensitivity analyses: (1) with data from studies that reported risk factors for ICU admission as the only poor outcome, (2) excluding studies with mortality as a poor outcome and (3) excluding studies with use of supplementary oxygen as a poor outcome. A list of outcomes used in each study and the list of studies included in each subgroup and sensitivity analysis are available in Table, Supplemental Digital Content 5, http://links.lww.com/INF/F406.

## RESULTS

### Characteristics of the Included Studies

A total of 30 studies met the selection criteria and were included in the systematic review. According to the UN classification, 17 studies were conducted in developing countries and 13 studies in developed countries (see Figure, Supplemental Digital Content 6, http://links.lww.com/INF/F407).^20^ A total of 66.7% of the included studies were retrospective and 33.3% were prospective in design. Additionally, 50.0% of the studies only included children under 2 years of age. Overall, the included studies summarized data on 173,990 children with ALRI. Of a maximum score of 14, the quality of the studies ranged from 5 to 11 with a median of 8 (see details in Table, Supplemental Digital Content 4, http://links.lww.com/INF/F405). Baseline characteristics of all the included studies are shown in detail in Table, Supplemental Digital Content 7, http://links.lww.com/INF/F408.

In our review, 27 of the included studies reported risk factors for poor outcomes among children with RSV-associated ALRI,^17,22–47^ 2 studies reported data for influenza,^48,49^ and 1 study for SARS-CoV-2.^50^ Therefore, we only carried out meta-analysis and subgroup analysis for RSV-associated ALRI. Meta-estimates were not possible for influenza or SARS-CoV-2. Instead, we used descriptive analysis to summarize the findings.

### Risk Factors for Poor Outcomes: Respiratory Syncytial Virus

For RSV-related ALRI, sufficient data were available to perform meta-analysis for 10 clinical and 5 sociodemographic risk factors. Results of the meta-analysis from studies with multivariable and univariable analyses are summarized in Table 1 (clinical risk factors) and Table 2 (sociodemographic risk factors).

**TABLE 1.** Results of the Meta-analysis, Sensitivity Analysis and Subgroup Analysis for Clinical Risk Factors of Poor Outcomes in Children Under 5 Years Old Hospitalized With Respiratory Syncytial Virus-associated Acute Lower Respiratory Infection

Risk Factor	Analysis Type		Studies With Multivariable Analysis			Studies With Univariable Analysis		
			No. of Studies	OR (95% CI)	*I*^2^, %	No. of Studies	OR (95% CI)	*I*^2^, %
Neurologic disease	Overall		4	6.14 (2.39–15.77)	82.2	4	5.40 (2.31–12.59)	71.0
	Sensitivity analysis:	QA score ≥7	4	6.14 (2.39–15.77)	82.2	3	4.76 (1.78–12.75)	80.6
		ICU admission only	2	NA		2	NA	
		Excl. mortality	4	6.14 (2.39–15.77)	82.2	3	4.76 (1.78–12.75)	80.6
		Excl. supplementary O_2_	3	5.53 (1.69–18.14)	89.1	3	5.56 (1.54–20.00)	76.7
	Subgroup analysis:	<2 years old	2	NA		2	NA	
		Developed	1	NA		2	NA	
		Developing	3	6.15 (1.57–24.05)	84.5	2	NA	
Down’s syndrome	Overall		4	5.43 (3.02–9.76)	14.2	4	9.21 (4.46–19.02)	55.1
	Sensitivity analysis:	QA score ≥7	4	5.43 (3.02–9.76)	14.2	3	6.69 (3.98–11.25)	0.0
		ICU admission only	1	NA		0	NA	
		Excl. mortality	4	5.43 (3.02–9.76)	14.2	3	6.69 (3.98–11.25)	0.0
		Excl. supplementary O_2_	2	NA		2	NA	
	Subgroup analysis:	<2 years old	2	NA		3	11.54 (4.46–29.86)	59.6
		Developed	2	NA		2	NA	
		Developing	2	NA		2	NA	
Chronic lung disease	Overall		5	3.64 (1.31–10.09)	73.9	8	2.56 (0.92–7.12)	90.7
	Sensitivity analysis:	QA score ≥7	2	NA		5	1.09 (0.46–2.60)	83.4
		ICU admission only	1	NA		2	NA	
		Excl. mortality	4	2.28 (0.97–5.35)	50.3	5	1.74 (0.56–5.34)	90.3
		Excl. supplementary O_2_	4	2.28 (0.97–5.35)	50.3	6	2.76 (0.86–8.89)	86.2
	Subgroup analysis:	<2 years old	2	NA		4	3.43 (0.56–20.96)	91.6
		Developed	1	NA		3	1.74 (0.15–19.67)	94.6
		Developing	4	3.72 (1.06–13.06)	80.2	5	3.56 (1.35–9.41)	81.8
Immunocompromised status	Overall		4	3.41 (1.85–6.29)	0.0	5	2.68 (1.62–4.44)	24.7
	Sensitivity analysis:	QA score ≥7	2	NA		2	NA	
		ICU admission only	1	NA		1	NA	
		Excl. mortality	3	3.08 (1.51–6.27)	2.8	3	2.08 (0.65–6.70)	64.0
		Excl. supplementary O_2_	3	3.08 (1.51–6.27)	2.8	3	2.08 (0.65–6.70)	64.0
	Subgroup analysis:	<2 years old	1	NA		2	NA	
		Developed	0	NA		1	NA	
		Developing	4	3.41 (1.85–6.29)	0.0	4	2.71 (1.50–4.92)	35.9
Prematurity (gestational age <37 weeks)	Overall		9	2.98 (1.93–4.59)	86.3	11	2.34 (1.71–3.20)	81.9
	Sensitivity analysis:	QA score ≥7	6	3.30 (1.89–5.78)	87.6	5	1.75 (1.07–2.85)	83.9
		ICU admission only	5	3.75 (1.74–8.08)	92.1	4	2.91 (2.51–3.37)	0.0
		Excl. mortality	8	3.16 (1.94–5.13)	88.3	8	2.53 (2.12–3.01)	38.4
		Excl. supplementary O_2_	5	3.75 (1.74–8.08)	92.1	8	2.56 (1.45–4.51)	92.7
	Subgroup analysis:	<2 years old	4	4.13 (1.88–9.08)	87.3	5	3.44 (1.77–6.68)	91.2
		Developed	4	2.96 (1.73–5.09)	71.6	5	2.60 (2.12–3.20)	19.2
		Developing	5	3.02 (1.49–6.10)	92.0	6	1.98 (1.09–3.60)	89.9
Congenital heart disease	Overall		8	2.80 (1.84–4.24)	75.5	8	3.05 (1.78–5.25)	87.8
	Sensitivity analysis:	QA score ≥7	5	3.12 (1.75–5.58)	76.1	6	2.55 (1.53–4.25)	71.7
		ICU admission only	5	3.60 (2.47–5.25)	47.1	3	3.69 (2.25–6.06)	49.4
		Excl. mortality	7	2.63 (1.69–4.09)	77.7	6	2.53 (1.62–3.96)	81.2
		Excl. supplementary O_2_	7	2.63 (1.69–4.09)	77.7	7	3.11 (1.67–5.82)	90.4
	Subgroup analysis:	<2 years old	2	NA		4	3.11 (1.07–9.07)	92.4
		Developed	2	NA		2	NA	
		Developing	6	2.50 (1.54–4.07)	79.3	6	2.56 (1.61–4.09)	81.4
Underlying disease	Overall		6	2.45 (1.94–3.09)	0.0	9	2.04 (1.48–2.82)	82.2
	Sensitivity analysis:	QA score ≥7	4	2.56 (1.99–3.28)	0.0	6	2.11 (1.33–3.34)	87.9
		ICU admission only	5	2.29 (1.63–3.20)	0.0	6	2.16 (1.46–3.19)	73.3
		Excl. mortality	6	2.45 (1.94–3.09)	0.0	8	2.29 (1.71–3.05)	75.1
		Excl. supplementary O_2_	6	2.45 (1.94–3.09)	0.0	9	2.04 (1.48–2.82)	82.2
	Subgroup analysis:	<2 years old	3	2.48 (1.86–3.30)	0.0	4	2.21 (1.55–3.16)	60.4
		Developed	3	2.35 (1.35–4.09)	37.5	3	1.76 (1.28–2.42)	0.0
		Developing	3	2.48 (1.86–3.30)	0.0	6	2.09 (1.36–3.23)	88.7
Viral coinfection	Overall		6	2.01 (1.27–3.19)	83.4	8	1.26 (0.78–2.03)	84.5
	Sensitivity analysis:	QA score ≥7	4	1.83 (0.87–3.87)	89.4	5	1.00 (0.60–1.67)	84.5
		ICU admission only	3	2.73 (1.78–4.20)	67.1	2	NA	
		Excl. mortality	6	2.01 (1.27–3.19)	83.4	6	1.47 (0.82–2.63)	87.8
		Excl. supplementary O_2_	4	2.37 (1.62–3.47)	76.2	5	1.40 (0.75–2.61)	89.7
	Subgroup analysis:	<2 years old	3	1.85 (1.39–2.46)	3.7	4	1.88 (1.16–3.06)	56.4
		Developed	2	NA		2	NA	
		Developing	4	2.37 (1.62–3.47)	76.2	6	1.30 (0.76–2.25)	86.2
Low birth weight (<2500 g)	Overall		3	1.88 (1.19–2.95)	0.0	2	NA	
	Sensitivity analysis:	QA score ≥7	1	NA		0	NA	
		ICU admission only	2	NA		1	NA	
		Excl. mortality	3	1.88 (1.19–2.95)	0.0	2	NA	
		Excl. supplementary O_2_	3	1.88 (1.19–2.95)	0.0	2	NA	
	Subgroup analysis:	<2 years old	2	NA		2	NA	
		Developed	1	NA		0	NA	
		Developing	2	NA		2	NA	
Underweight	Overall		5	1.80 (1.38–2.35)	33.0	5	2.45 (1.56–3.84)	78.2
	Sensitivity analysis:	QA score ≥7	3	1.68 (1.23–2.30)	29.0	3	2.00 (1.19–3.36)	79.3
		ICU admission only	3	2.15 (1.18–3.92)	61.3	3	2.36 (1.19–4.69)	85.5
		Excl. mortality	4	1.97 (1.33–9.37)	51.2	4	2.34 (1.44–3.80)	82.9
		Excl. supplementary O_2_	3	2.15 (1.18–3.92)	61.3	3	2.36 (1.19–4.69)	85.5
	Subgroup analysis:	<2 years old	3	1.55 (1.29–1.86)	0.0	3	1.98 (1.15–3.42)	79.0
		Developed	2	NA		2	NA	
		Developing	3	1.90 (1.38–2.63)	0.0	3	2.64 (1.88–3.71)	0.0

ICU indicates intensive care unit; NA, not available; QA, quality assessment.

**TABLE 2.** Results of the Meta-analysis, Sensitivity Analysis and Subgroup Analysis for Sociodemographic Risk Factors of Poor Outcomes in Children Under 5 Years Old Hospitalized With Respiratory Syncytial Virus-associated Acute Lower Respiratory Infection

Risk Factor	Analysis Type		Studies With Multivariable Analysis			Studies With Univariable Analysis		
			No. of Studies	OR (95% CI)	*I*^2^, %	No. of Studies	OR (95% CI)	*I*^2^, %
Age <2 months	Overall		4	2.29 (1.78–2.94)	6.6	3	1.82 (1.01–3.29)	81.5
	Sensitivity analysis:	QA score ≥7	3	2.13 (1.62–2.81)	1.0	3	1.82 (1.01–3.29)	81.5
		ICU admission only	2	NA		1	NA	
		Excl. mortality	4	2.29 (1.78–2.94)	6.6	3	1.82 (1.01–3.29)	81.5
		Excl. supplementary O_2_	3	2.40 (1.50–3.83)	45.1	2	NA	
	Subgroup analysis:	<2 years old	2	NA		2	NA	
		Developed	3	2.57 (1.94–3.41)	0.0	2	NA	
		Developing	1	NA		1	NA	
Age <6 months	Overall		6	2.08 (1.81–2.39)	0.0	5	2.37 (2.00–2.82)	0.0
	Sensitivity analysis:	QA score ≥7	4	2.34 (1.76–3.11)	0.0	4	2.35 (1.97–2.81)	0.0
		ICU admission only	3	2.07 (1.77–2.42)	0.0	1	NA	
		Excl. mortality	5	2.09 (1.81–2.42)	0.0	4	2.35 (1.97–2.81)	0.0
		Excl. supplementary O_2_	3	2.07 (1.77–2.42)	0.0	2	NA	
	Subgroup analysis:	<2 years old	2	NA		2	NA	
		Developed	2	NA		2	NA	
		Developing	4	2.04 (1.76–2.37)	0.0	3	2.66 (1.57–4.50)	51.2
Male sex	Overall		6	1.15 (0.98–1.35)	0.8	12	1.09 (0.97–1.22)	31.1
	Sensitivity analysis:	QA score ≥7	5	1.19 (1.00–1.40)	0.0	8	1.09 (0.94–1.27)	53.3
		ICU admission only	4	1.18 (0.94–1.47)	0.0	6	1.02 (0.91–1.14)	0.0
		Excl. mortality	6	1.15 (0.98–1.35)	0.8	10	1.10 (0.98–1.40)	30.6
		Excl. supplementary O_2_	5	1.10 (0.92–1.31)	0.0	10	1.05 (0.94–1.19)	30.1
	Subgroup analysis:	<2 years old	4	1.12 (0.88–1.43)	26.6	6	1.06 (0.88–1.28)	31.1
		Developed	1	NA		2	NA	
		Developing	5	1.16 (0.97–1.38)	8.5	10	1.09 (0.96–1.24)	41.0
Lack of breastfeeding	Overall		0	NA		3	1.66 (1.24–2.24)	0.0
	Sensitivity analysis:	QA score ≥7	0	NA		2	NA	
		ICU admission only	0	NA		1	NA	
		Excl. mortality	0	NA		2	NA	
		Excl. supplementary O_2_	0	NA		1	NA	
	Subgroup analysis:	<2 years old	0	NA		3	1.66 (1.24–2.24)	0.0
		Developed	0	NA		0	NA	
		Developing	0	NA		3	1.66 (1.24–2.24)	0.0
Smoke exposure	Overall		2	NA		3	1.08 (0.83–1.41)	46.5
	Sensitivity analysis:	QA score ≥7	2	NA		2	NA	
		ICU admission only	1	NA		1	NA	
		Excl. mortality	2	NA		3	1.08 (0.83–1.41)	46.5
		Excl. supplementary O_2_	1	NA		2	NA	
	Subgroup analysis:	<2 years old	2	NA		3	1.08 (0.83–1.41)	46.5
		Developed	0	NA		0	NA	
		Developing	2	NA		3	1.08 (0.83–1.41)	46.5

ICU indicates intensive care unit; NA, not available; QA, quality assessment.

#### Meta-analysis of Clinical Risk Factors for Respiratory Syncytial Virus-Acute Lower Respiratory Infection Poor Outcomes

Neurologic disease, Down’s syndrome, chronic lung disease, immunocompromised status, prematurity (defined as gestational age <37 weeks), congenital heart disease, underlying disease, viral coinfection with any respiratory virus, low birth weight (<2500 g) and being underweight were reported with OR meta-estimates of 6.14 (95% CI: 2.39–15.77), 5.43 (95% CI: 3.02–9.76), 3.64 (95% CI: 1.31–10.09), 3.41 (95% CI: 1.85–6.29), 2.98 (95% CI: 1.93 – 4.59), 2.80 (95% CI: 1.84–4.24), 2.45 (95% CI: 1.94–3.09), 2.01 (95% CI: 1.27–3.19), 1.88 (95% CI: 1.19–2.95) and 1.80 (95% CI: 1.38–2.35), respectively (based on studies with multivariable data). Subgroup analysis by age group and world region, as well as sensitivity analysis with ICU admission as the only poor outcome showed similar results. When studies with mortality as a poor outcome and studies with use of supplementary oxygen as a poor outcome were excluded in separate sensitivity analyses, the meta-estimates were similar except for chronic lung disease (OR: 2.28; 95% CI: 0.97–5.35) versus 3.64 (95% CI: 1.31–10.09) for both sensitivity analyses. Sensitivity analysis based on quality assessment scores of 7 and above had similar results except for viral coinfection (OR: 1.83; 95% CI: 0.87–3.87). More details are available in Table 1.

#### Meta-analysis of Sociodemographic Risk Factors for Respiratory Syncytial Virus-Acute Lower Respiratory Infection Poor Outcomes

Age <2 months, age <6 months and male sex were reported with OR meta-estimates of 2.29 (95% CI: 1.78–2.94), 2.08 (95% CI: 1.81–2.39 and 1.15 (95% CI: 0.98–1.35), respectively (based on studies with multivariable data). Sensitivity analyses had similar estimates. Based on univariable analysis, OR meta-estimates for the lack of breastfeeding and exposure to smoke were 1.66 (95% CI: 1.24–2.24) and 1.08 (95% CI: 0.83–1.41), respectively. More details are shown in Table 2.

#### Other Risk Factors for Respiratory Syncytial Virus-Acute Lower Respiratory Infection Poor Outcomes

Several other risk factors for poor outcomes in young children hospitalized with RSV-ALRI were reported; however, due to the small number of studies reporting these risk factors, no meta-analyses were performed. Eight clinical risk factors for poor outcomes were identified in our list of included studies. Gastrointestinal diseases were reported as significant risk factors in 2 studies using multivariable analysis with ORs of 14.99–15.01.^22,24^ Hematologic conditions were identified as a significant risk factor for poor outcomes in 1 study based on multivariable analysis with OR 3.67.^24^ Metabolic conditions were reported as a significant risk factor for poor outcomes in 1 study using univariable analysis with OR 6.70.^35^ Vitamin D deficiency was identified as a risk factor in 2 studies using multivariable analysis, with ORs of 1.94–9.02 (1 OR was significant).^24,30^ Two studies reported being overweight as a risk factor for poor outcomes using multivariable analyses, but the OR meta-estimates were not significant.^37,47^ Chronic kidney disease was reported in 1 study using multivariable analysis with OR of 1.54 (95% CI: 0.70–3.40).^40^ Incomplete immunization for age was reported in 1 study based on multivariable analysis with OR 1.95 for pneumococcal conjugate vaccine and OR 2.48 for influenza vaccine (the former was significant).^41^ Family history of atopy was reported in 3 studies based on univariable analysis with ORs 0.61–1.22.^27,28,46^

Additionally, 6 sociodemographic risk factors for poor outcomes were identified. Low socio-economic status was reported as a significant risk factor in 1 study based on multivariable analysis with OR 1.56.^41^ Over-crowding was reported in 2 studies using univariable analysis but the ORs were not significant.^28,46^ Additionally, 1 study identified a significant association between over-crowding and poor outcomes in male children with OR 2.36 in multivariable analysis; however, for female children the OR was not significant.^27^ Precarious home, defined as no sewage system at home or household use of wood as a cooking fuel, was identified as a significant risk factor in 2 studies based on multivariable analyses with ORs 1.66–1.72.^28,39^ Vulnerable mother, living far from hospital and indigenous ethnicity were each reported in 1 study, but were not significant risk factors.^28,41^

#### Post-discharge Complications in Children With Respiratory Syncytial Virus-Acute Lower Respiratory Infection Poor Outcomes

Three studies reported follow-up data on hospital readmissions in children who had experienced poor outcomes due to RSV-ALRI. Univariable data from 1 retrospective study conducted in Israel reported chronic comorbidities as a significant risk factor for hospital readmission due to respiratory disease during the study period with OR 2.86 (95% CI: 1.05–7.76).^42^ A retrospective study from Spain found several risk factors to be significantly associated with hospital readmission within 30 days after discharge based on univariable data: prematurity (OR: 3.66; 95% CI: 3.15–4.27), congenital heart disease (OR: 3.05; 95% CI: 2.51–3.71), chronic lung disease (OR: 6.76; 95% CI: 3.87–11.83), Down’s syndrome (OR: 2.65; 95% CI: 1.83–3.84), neuromuscular disorders (OR: 5.42; 95% CI: 3.72–7.89) and immunodeficiency (OR: 4.35; 95% CI: 1.84–10.30).^17^ One retrospective study from Japan reported data on hospital readmissions in underweight and overweight children with OR 1.12 and OR 0.95, respectively (none of the ORs were significant).^37^

### Risk Factors for Poor Outcomes: Influenza

One retrospective case study, which was carried out in Colombia and included data on 535 children under 2 years old, reported risk factors for influenza-related ALRI poor outcomes. The composite OR for conditions identified by Advisory Committee for Immunization Practices (ACIP) as high-risk conditions for influenza complications, including chronic cardiovascular disease, chronic lung disease, asthma, metabolic disorders, endocrine disorders, immunosuppression, aspirin therapy, hemoglobinopathies and renal disease, were reported separately from the composite OR of all other chronic diseases.^48^ In the multivariable model adjusted for age and social security system affiliation scheme, ORs for mechanical ventilation and mortality were 7.40 (95% CI: 2.90–19.30) and 30.0 (95% CI: 7.40–120.90) respectively for children with ACIP identified conditions who were hospitalized for influenza. For children with non-ACIP-identified conditions compared to their peers, ORs for mechanical ventilation and mortality were 3.10 (95% CI: 1.20–8.50) and 10.60 (95% CI: 2.60–42.90), respectively.^48^ In addition, a cross-sectional study conducted in the United States reported more severe in-hospital outcomes in older children: compared to children under 6 months old, age 6–24 months was identified as a risk factor for ICU admission with OR 1.29 (95% CI: 1.11–1.51) and mechanical ventilation with OR 1.16 (95% CI: 0.88–1.53) based on multivariable analysis adjusted for study site, influenza testing and test sensitivity.^49^

### Risk Factors for Poor Outcomes: Severe Acute Respiratory Syndrome Coronavirus 2

One retrospective cross-sectional study from Mexico reported risk factors for mortality in children under 18 years old with SARS-CoV-2 related ALRI. For the cases (children who died) and the control group (children who did not die), oxygen therapy status and other poor outcome indicators were unclear. The study identified risk factors for mortality in a subpopulation of 257 children under 1-year of age (94.2% hospitalized children and 5.8% outpatients). In children under 1-year-old, significant risk factors for mortality were cardiovascular disease (OR: 13.85; 95% CI: 7.80–24.30), immunosuppression (OR: 7.02; 95% CI: 3.10–15.50), high blood pressure (OR: 4.81; 95% CI: 3.10–7.30), chronic kidney disease (OR: 4.80; 95% CI: 1.31–17.50) and diabetes (OR: 4.41; 95% CI: 2.60–7.30) based on multivariable analysis.^50^

## DISCUSSION

Our comprehensive systematic review of data from 173,990 children hospitalized with ALRI has identified that underlying chronic conditions, including heart disease, immunosuppression and chronic lung disease, remain significant risk factors for poor outcomes associated with 3 major respiratory viral infections of childhood including RSV, influenza and SARS-CoV-2. Looking at risk factors for severe disease among hospitalized patients is useful for clinicians dealing with this population as it might help to identify early on those that will require an escalation of care and more intensive use of resources. There is also potential value from a population-level perspective to identify high-risk groups to prioritize for preventive efforts such as vaccination. Although the Pfizer-BioNTech and Moderna COVID-19 vaccines have only received Food and Drug Administration (FDA) approval for emergency use in children 6 months to 4 years old, there is an effective vaccine against influenza which is specifically recommended for children ≥6 months of age with chronic medical conditions. However, vaccine uptake in children with chronic lung diseases remains low, which is not acceptable for other pediatric vaccines.^51,52^ Our results further highlight the need for enhanced strategies to improve influenza vaccine uptake in children with chronic diseases.

Our meta-analysis identified a total of 12 significant risk factors for RSV-ALRI associated poor outcomes. The meta-estimates suggest the highest risk of RSV-ALRI poor outcomes among children with neurologic disease (OR: 6.14; 95% CI: 2.39–15.77) and Down’s syndrome (OR: 5.43; 95% CI: 3.02–9.76). A previous estimate has also suggested that children with Down’s syndrome who are hospitalized with RSV-ALRI are at more than 6 times higher risk (OR: 6.53; 95% CI: 2.22–19.19) of requiring oxygen support (a marker for poor outcomes) compared to children without Down’s syndrome.^16^ The increased risk in children with Down’s syndrome has been linked to high prevalence of congenital heart disease and pulmonary hypertension and has also been attributed to airway abnormality, injury-prone lung and hypotonia in the absence of underlying chronic conditions.^53–55^ Our meta-analyses suggest the need for prevention of severe RSV disease in children with Down’s syndrome. However, the American Academy of Pediatrics does not recommend routine use of palivizumab, the only currently available RSV prophylaxis, in children with Down’s syndrome, highlighting the need for revisiting clinical practice guidelines for RSV prophylaxis.^56^

For RSV-ALRI, we identified congenital heart disease to significantly increase the risk of poor outcomes (OR: 2.80; 95% CI: 1.84–4.24). This finding is consistent with a previous study (OR: 3.40; 95% CI: 2.14–5.40).^14^ We also found chronic lung disease to be a significant risk factor for poor outcomes with OR 3.64 (95% CI: 1.31–10.09), which is consistent with previous findings of bronchopulmonary dysplasia as significantly associated with ICU admission (OR: 2.90; 95% CI: 2.30–3.50).^15^ Age <2 months and <6 months at the time of hospitalization were identified as risk factors for RSV-ALRI poor outcomes in multivariable analysis with OR: 2.29 (95% CI: 1.78–2.94) and OR: 2.08 (95% CI: 1.81–2.39), respectively. Increased risk of poor outcomes among children <6 months old has been reported previously with OR: 2.02 (95% CI: 1.73–2.35).^14^ Smaller caliber airways and less mature immune systems contribute to the development of more severe ALRI in young children.^31,57^ Our meta-analysis suggests that male sex is not significantly associated with poor outcomes (OR: 1.15; 95% CI: 0.98–1.35), which corresponds to a previous study with OR meta-estimate 1.39 (95% CI: 0.95–2.04).^14^

This study has several notable strengths. Our review investigates risk factors associated with poor outcomes for 3 common respiratory viruses, provides a large total sample size of 173,990 children, and reports many risk factors. Additionally, the subgroup meta-analyses stratified by region and age group allow for evaluation of the findings for specific subpopulations. The studies included in this review were published between 2011 and 2023; therefore, our review reflects more recent evidence than in previous reviews.^14,15,58^ RSV preventive therapeutics (eg, extended half-life mAb, maternal and childhood vaccines) for children are increasingly available: a mAb has been approved for infants by the United Kingdom Medicines and Healthcare products Regulatory Agency, European Medicines Agency and US FDA, and maternal vaccination has been approved by FDA. Additional products will become available soon. This progress makes this paper timely to help stratify the roll-out of passive immunization by at risk group—leading to the increased importance of these data for public health policy.

However, this study has several limitations. First, there was insufficient data to conduct meta-analysis of risk factors for poor outcomes in cases of ALRI related to influenza and SARS-CoV-2. Although there are many studies on severe outcomes among hospitalized children for both infections, there is still limited knowledge on the risk factors for the severe outcomes. More research into the severity of these viral infections in young children is needed.

Second, due to limited data, ORs and RRs were combined in the meta-analysis to provide rough estimates possibly reducing the reliability of the meta-estimates. Limited data were available for subgroup analysis focusing on each category of poor outcome and the 95% CIs were broad in general. The risk factors for poor outcomes related to RSV infection reported in less than 3 studies illustrate areas for future research. In this review, we did not find any data on other potential outcomes (in-hospital complications) such as sepsis, apneic episodes and pleural effusion, highlighting a knowledge gap in this field. It would be beneficial to conduct additional literature searches in languages other than English.

Third, there was a lack of consistency in the definitions of risk factors and outcomes across studies; therefore, similar definitions were grouped together to perform meta-analyses (see Table, Supplemental Digital Content 3, http://links.lww.com/INF/F404). Prolonged hospital stay was defined as 5 or more days, as this was common in the included studies; however, studies reporting longer hospital stays were also included in this definition. Additionally, more data would be needed to analyze the risk factors in nuance, for instance to determine the strength of associations between specific congenital heart diseases and poor outcomes.

Fourth, the available medical facilities varied across studies and had varying thresholds for interventions. A total of 81% of deaths in children with severe ALRI are estimated to take place outside of hospitals.^59^ Therefore, population-based studies are needed to include children with severe disease in rural areas or without access to health care facilities. Prospective surveillance studies are necessary to account for all risk factors of interest. The treating physicians might have been inclined to focus interventions on children they considered at higher risk. This could result in some risk factors being overestimated with poor outcomes.^38^

Fifth, as demonstrated in the quality assessment, the lack of matched controls, use of univariable analysis, retrospective study design and other disadvantages may introduce bias and reduce the reliability of the results. Although the median score of the quality assessment was 8, to have enough studies for meta-analysis, sensitivity analysis included all studies with a score of 7 and above. Twenty of the 30 included studies were retrospective, possibly leading to exclusion of children due to misclassified or incomplete data. To increase the accuracy of the meta-estimates, multivariable analysis should be used and adjusted for the same confounders across all studies. In addition, there might be a concern about admission (collider) bias as underlying medical conditions can affect both the likelihood of hospitalization and of severe outcomes. This can preclude generalizability beyond the hospital setting and the interpretation of results should be done with great care.

Further studies should examine the breadth of ALRI with RSV, influenza and SARS-CoV-2 and include asymptomatic carriage in the community, which may provide better understanding of the impact of RSV infection on hospital outcomes in the general population. Moreover, using nonhospitalized community controls would be helpful to elucidate the risk profiles which put underlying populations at higher risk of hospital admission with any of these respiratory viruses.

In conclusion, as RSV prophylactics become part of routine clinical care, the data from this study will help in assessment of real-world effectiveness of RSV prophylactics in reducing short and long-term complications. Additionally, the data may also contribute to the development of guidelines for the use of influenza and SARS-CoV-2 vaccinations in children at high risk for severe outcomes due to ALRI.

## Supplementary Material


