# Supplementary material for: Risk Factors for Poor Outcomes in Children Hospitalized With Virus-associated Acute Lower Respiratory Infections: A Systematic Review and Meta-analysis
Source: Pediatr Infect Dis J. 2024 Jan 26;43(5):467–76. doi: 10.1097/INF.0000000000004258 (PMC11003409; doi:10.1097/INF.0000000000004258)
Supplement: Supplementary file 1 [file inf-43-0467-s001.docx]

**Supplemental Digital Content 1.** Search strategies.

| **Database: Embase** | |
| --- | --- |
| **Search strategy** | **Results** |
| 1 respiratory tract infections.mp.  2 bacterial pneumonia/ or community acquired pneumonia/ or virus pneumonia/ or hospital acquired pneumonia/ or pneumonia.mp.  3 bronchiolitis.mp.  4 viral bronchiolitis.mp.  5 alri.mp.  6 acute lower respiratory infection.mp.  7 acute lower respiratory tract infection.mp.  8 lower respiratory infection.mp.  9 lower respiratory tract infection.mp.  10 1 or 2 or 3 or 4 or 5 or 6 or 7 or 8 or 9  11 risk factor.mp.  12 exp risk/  13 predisposing factor.mp. or exp predisposing factor/  14 disease predisposition.mp. or exp disease predisposition/  15 disease susceptibility.mp. or exp disease susceptibility/  16 predictor.mp.  17 predictor variable.mp.  18 11 or 12 or 13 or 14 or 15 or 16 or 17  19 hospitalization.mp. or exp hospitalization/  20 hospital admission.mp.  21 length of stay.mp.  22 length of hospital stay.mp.  23 days in hospital.mp.  24 19 or 20 or 21 or 22 or 23  25 intensive care unit.mp. or exp intensive care unit/  26 critical care unit.mp. or exp critical care unit/  27 mechanical ventilation.mp. or exp mechanical ventilation/  28 oxygen supplementation.mp. or exp oxygen supplementation  29 intubation.mp. or exp intubation/  30 artificial ventilation.mp. or exp artificial ventilation/  31 artificial respiration.mp. or exp artificial respiration/  32 noninvasive ventilation.mp. or exp noninvasive ventilation/  33 hospital mortality.mp. or exp hospital mortality/  34 in-hospital mortality.mp. or exp in-hospital mortality/  35 exp death/  36 mortality.mp. or exp mortality/  37 poor outcome.mp.  38 severe disease.mp.  39 critically ill.mp. or exp critically ill patient/  40 25 or 26 or 27 or 28 or 29 or 30 or 31 or 32 or 33 or 34 or 35 or 36 or 37 or 38 or 39  41 10 and 18 and 24 and 40  42 limit 41 to (english language and yr="2011 -Current" and (infant <to one year> or preschool child <1 to 6 years>)) | 606 |
| **Database: Medline** | |
| **Search strategy** | **Results** |
| 1 respiratory tract infections.mp.  2 bacterial pneumonia/ or community acquired pneumonia/ or virus pneumonia/ or hospital acquired pneumonia/ or pneumonia.mp.  3 bronchiolitis.mp.  4 viral bronchiolitis.mp.  5 alri.mp.  6 acute lower respiratory infection.mp.  7 acute lower respiratory tract infection.mp.  8 lower respiratory infection.mp.  9 lower respiratory tract infection.mp.  10 1 or 2 or 3 or 4 or 5 or 6 or 7 or 8 or 9  11 risk factor.mp.  12 exp risk/  13 predisposing factor.mp. or exp predisposing factor/  14 disease predisposition.mp. or exp disease predisposition/  15 disease susceptibility.mp. or exp disease susceptibility/  16 predictor.mp.  17 predictor variable.mp.  18 11 or 12 or 13 or 14 or 15 or 16 or 17  19 hospitalization.mp. or exp hospitalization/  20 hospital admission.mp.  21 length of stay.mp.  22 length of hospital stay.mp.  23 days in hospital.mp.  24 19 or 20 or 21 or 22 or 23  25 intensive care unit.mp. or exp intensive care unit/  26 critical care unit.mp. or exp critical care unit/  27 mechanical ventilation.mp. or exp mechanical ventilation/  28 oxygen supplementation.mp. or exp oxygen supplementation/  29 intubation.mp. or exp intubation/  30 artificial ventilation.mp. or exp artificial ventilation/  31 artificial respiration.mp. or exp artificial respiration/  32 noninvasive ventilation.mp. or exp noninvasive ventilation/  33 hospital mortality.mp. or exp hospital mortality/  34 in-hospital mortality.mp. or exp in-hospital mortality/  35 exp death/  36 mortality.mp. or exp mortality/  37 poor outcome.mp.  38 severe disease.mp.  39 critically ill.mp. or exp critically ill patient/  40 25 or 26 or 27 or 28 or 29 or 30 or 31 or 32 or 33 or 34 or 35 or 36 or 37 or 38 or 39  41 10 and 18 and 24 and 40  42 limit 41 to (english language and yr="2011 -Current" and ("all infant (birth to 23 months)" or "preschool child (2 to 5 years)")) | 486 |
| **Database: Global Health** | |
| **Search strategy** | **Results** |
| 1 respiratory tract infections.mp. [mp=abstract, title, original title, heading words, cabicodes words]  2 bacterial pneumonia/ or community acquired pneumonia/ or virus pneumonia/ or hospital acquired pneumonia/ or pneumonia.mp.  3 bronchiolitis.mp.  4 viral bronchiolitis.mp.  5 alri.mp.  6 acute lower respiratory infection.mp.  7 acute lower respiratory tract infection.mp.  8 lower respiratory infection.mp.  9 lower respiratory tract infection.mp.  10 1 or 2 or 3 or 4 or 5 or 6 or 7 or 8 or 9  11 risk factor.mp.  12 exp risk/  13 predisposing factor.mp.  14 disease predisposition.mp.  15 disease susceptibility.mp.  16 predictor.mp.  17 predictor variable.mp.  18 11 or 12 or 13 or 14 or 15 or 16 or 17  19 hospitalization.mp.  20 hospital admission.mp.  21 length of stay.mp.  22 length of hospital stay.mp.  23 days in hospital.mp.  24 19 or 20 or 21 or 22 or 23  25 intensive care unit.mp. or exp intensive care unit/  26 critical care unit.mp.  27 mechanical ventilation.mp.  28 oxygen supplementation.mp.  29 intubation.mp.  30 artificial ventilation.mp. or exp artificial ventilation/  31 artificial respiration.mp. or exp artificial respiration/  32 noninvasive ventilation.mp.  33 hospital mortality.mp.  34 in-hospital mortality.mp.  35 exp death/  36 mortality.mp. or exp mortality/  37 poor outcome.mp.  38 severe disease.mp.  39 critically ill.mp.  40 25 or 26 or 27 or 28 or 29 or 30 or 31 or 32 or 33 or 34 or 35 or 36 or 37 or 38 or 39  41 10 and 18 and 24 and 40  42 limit 41 to (english language and yr="2011 -Current") | 511 |
