# Supplementary material for: Risk Factors for Poor Outcomes in Children Hospitalized With Virus-associated Acute Lower Respiratory Infections: A Systematic Review and Meta-analysis
Source: Pediatr Infect Dis J. 2024 Jan 26;43(5):467–76. doi: 10.1097/INF.0000000000004258 (PMC11003409; doi:10.1097/INF.0000000000004258)
Supplement: Supplementary file 2 [file inf-43-0467-s002.docx]

**Supplemental Digital Content 2.** Inclusion and exclusion criteria for study screening.

| **Inclusion criteria** | **Exclusion criteria** |
| --- | --- |
| Patients were children up to the age of 5 hospitalized with the diagnosis of acute lower respiratory tract infections. | Published before 2011. |
| Published from January 1, 2011 to January 6, 2023. | Published in languages other than English. |
| Published in English. | Articles irrelevant to the topic. |
| Original research articles, and publications that provide original data. | Publications with no original data. |
| Studies that report the risk factors for poor outcomes of acute lower respiratory tract infections. | Publications with unclear definitions (for risk factors or poor outcomes) or unclear methods for data analysis. |
| Focus on children with confirmed RSV, influenza, or SARS-CoV-2 infections. | Studies with patients without confirmed RSV, influenza, or SARS-CoV-2 infections. |
|  | Sample size less than 50. |
