# Supplementary material for: Risk Factors for Poor Outcomes in Children Hospitalized With Virus-associated Acute Lower Respiratory Infections: A Systematic Review and Meta-analysis
Source: Pediatr Infect Dis J. 2024 Jan 26;43(5):467–76. doi: 10.1097/INF.0000000000004258 (PMC11003409; doi:10.1097/INF.0000000000004258)
Supplement: Supplementary file 3 [file inf-43-0467-s003.docx]

**Supplemental Digital Content 3.** Definitions of the risk factors for poor outcomes in children with RSV-related ALRI used in the studies included in the systematic review and meta-analysis.

| **Risk factor** | **Definition** |
| --- | --- |
| Family history of atopy | - parent with asthma or atopic disease |
| Lack of breastfeeding | - lack of breastfeeding |
| Congenital heart disease | - cyanotic heart disease, - non-cyanotic heart disease, - cardiovascular disease, - congenital heart defects, - pulmonary hypertension, - arrhythmias, - myocardiopathy |
| Chronic kidney disease | - renal disease |
| Chronic lung disease | - chronic lung disease, - respiratory comorbidity, - pre-existing respiratory disease, - bronchopulmonary dysplasia, - asthma, - bronchomalacia, - malformations of the respiratory system |
| Viral coinfection | - evidence of any respiratory viral coinfection (e.g., parainfluenza type 3, adenovirus) |
| Down’s syndrome | - Down’s syndrome |
| Living far from hospital | - distance to the hospital > 30 min |
| Gastrointestinal disease | - gastrointestinal disease (e.g., cirrhosis, liver disease, biliary atresia) |
| Hematologic conditions | - blood disease (e.g., anaemias, haemorrhagic conditions) |
| Immunocompromised status | - immunodeficiency, - immunosuppression, - chemotherapy, - cancer, - human immunodeficiency virus (HIV), - exposure to HIV |
| Incomplete immunisation for age | - incomplete annual influenza immunisation for age, - incomplete PCV13 immunisation for age |
| Indigenous ethnicity | - indigenous ethnicity |
| Low socioeconomic status | - low social class (based on socioeconomic factors) |
| Low birth weight | - birth weight <2500 g |
| Metabolic conditions | - metabolic conditions (e.g., phenylketonuria, adrenal insufficiency) |
| Male sex | - male sex |
| Neurologic disease | - neurologic disease, - cerebral palsy, - neuromuscular disorders |
| Over-crowding | - more than 3 people in the same room, - family size of four or more, - seven or more household members |
| Overweight | - weight-for-age Z-score > 1, - weight-for-length > 97.7^th^ percentile (+2 SD of Z-score) |
| Prematurity | - born at gestational age <37 weeks |
| Precarious home | - no sewage system at home, - household use of wood as a cooking fuel |
| Smoke exposure | - tobacco smoking at home, - smoke exposure, - tobacco exposure |
| Underlying disease | - any underlying disease/ comorbidity (e.g., congenital heart disease, chronic lung disease, Down syndrome, congenital defects, prematurity) |
| Underweight | - weight-for-age Z-score < -2SD, - weight-for-age Z-score < -1SD, - weight-for-length < 5th percentile |
| Vitamin D deficiency | - vitamin D deficiency |
| Vulnerable mother | - adolescent mother (<18 y.o.), - late child-bearing (> 35 y.o.), - incomplete primary education |
| Young age | - <2 months, - <6 months, - 6 months-24 months |
