# Supplementary material for: Risk Factors for Poor Outcomes in Children Hospitalized With Virus-associated Acute Lower Respiratory Infections: A Systematic Review and Meta-analysis
Source: Pediatr Infect Dis J. 2024 Jan 26;43(5):467–76. doi: 10.1097/INF.0000000000004258 (PMC11003409; doi:10.1097/INF.0000000000004258)
Supplement: Supplementary file 4 [file inf-43-0467-s004.docx]

**Supplemental Digital Content 4**. Quality assessment.

| Study | Study design | Control group | Sample size | Analysis type | Avoiding bias | Confounding factors | Geographical spread | **Total** |
| --- | --- | --- | --- | --- | --- | --- | --- | --- |
| Aikphaibul *et al.* (2021) | 1 | 0 | 1 | 1 | 1 | 1 | 0 | **5** |
| Anderson *et al.* (2022) | 1 | 0 | 2 | 1 | 1 | 2 | 0 | **7** |
| Cai *et al.* (2020) | 1 | 1 | 2 | 2 | 1 | 1 | 2 | **10** |
| Chi *et al.* (2011) | 1 | 1 | 2 | 1 | 0 | 1 | 2 | **8** |
| Cotes *et al.* (2012) | 1 | 1 | 2 | 1 | 1 | 2 | 1 | **9** |
| Eski *et al.* (2021) | 1 | 1 | 2 | 2 | 1 | 2 | 0 | **9** |
| Ferolla *et al.* (2019) | 2 | 0 | 1 | 1 | 1 | 1 | 0 | **6** |
| Geoghegan *et al.* (2017) | 2 | 1 | 2 | 1 | 1 | 2 | 1 | **10** |
| Greenberg *et al.* (2014) | 2 | 0 | 2 | 2 | 1 | 1 | 1 | **9** |
| Halasa *et al.* (2015) | 2 | 1 | 2 | 2 | 1 | 1 | 0 | **9** |
| Helfrich *et al.* (2015) | 1 | 1 | 2 | 2 | 1 | 1 | 2 | **10** |
| Hervas *et al.* (2012) | 1 | 0 | 2 | 2 | 0 | 2 | 0 | **7** |
| Kamidani *et al.* (2022) | 1 | 1 | 2 | 2 | 1 | 1 | 2 | **10** |
| Fischer Langley *et al.* (2013) | 2 | 1 | 2 | 0 | 1 | 0 | 1 | **7** |
| Lu *et al.* (2015)^15^ | 1 | 0 | 1 | 2 | 1 | 1 | 0 | **6** |
| Martinez-Valdez *et al.* (2022) | 0.5 | 0 | 2 | 1 | 1 | 0 | 2 | **6.5** |
| Meenaghan *et al.* (2020) | 1 | 1 | 2 | 1 | 0 | 1 | 0 | **6** |
| Moreno-Perez et al. (2014) | 2 | 1 | 2 | 1 | 2 | 1 | 2 | **11** |
| Moyes *et al.* (2013) | 2 | 1 | 2 | 1 | 0 | 1 | 1 | **8** |
| Okubo *et al. (*2018) | 1 | 1 | 2 | 2 | 1 | 1 | 2 | **10** |
| Papenburg *et al.* (2012) | 2 | 1 | 1 | 1 | 1 | 1 | 1 | **8** |
| Patel *et al.* (2019) | 2 | 1 | 0 | 1 | 1 | 1 | 0 | **6** |
| Rodriguez *et al.* (2014) | 1 | 0 | 2 | 2 | 0 | 1 | 0 | **6** |
| Rodriguez-Martinez *et al.* (2022) | 0.5 | 1 | 2 | 2 | 1 | 1 | 2 | **9.5** |
| Sanchez-Luna *et al.* (2016) | 1 | 1 | 2 | 0 | 0 | 0 | 2 | **6** |
| Shmueli *et al.* (2021) | 1 | 1 | 2 | 2 | 1 | 2 | 0 | **9** |
| Stagliano *et al.* (2015) | 1 | 1 | 2 | 2 | 1 | 1 | 2 | **10** |
| Van de Steen *et al.* (2016) | 1 | 1 | 2 | 0 | 0 | 0 | 2 | **6** |
| Viguria *et al*. (2018) | 1 | 1 | 2 | 2 | 1 | 1 | 0 | **8** |
| Zhang *et al.* (2014) | 1 | 0 | 2 | 1 | 1 | 1 | 0 | **6** |

Modified Grading of Recommendations, Assessment, Development and Evaluations (GRADE) scoring system:

**Study design**: cohort study (2 points), case control study (1 point), cross sectional study (0.5 points), randomised control trial (0 points).

**Control group:** matched to cases (2), not matched but description of control group included (1), no detail on control group (0).

**Sample size:** >500 patients (2), 300-500 patients (1), <300 patients (0).

**Analysis type:** only multivariable (2), partially multivariable (1), only univariable (0).

**Avoiding bias:** good attempt (2), some attempt (1), no attempt (0).

**Confounding factors:** fully accounted for (2), some accounted for (1), not accounted for (0).

**Geographical spread of studies:** good (2), limited (1), no spread – only one region (0).
