# Supplementary material for: Risk Factors for Poor Outcomes in Children Hospitalized With Virus-associated Acute Lower Respiratory Infections: A Systematic Review and Meta-analysis
Source: Pediatr Infect Dis J. 2024 Jan 26;43(5):467–76. doi: 10.1097/INF.0000000000004258 (PMC11003409; doi:10.1097/INF.0000000000004258)
Supplement: Supplementary file 5 [file inf-43-0467-s005.docx]

**Supplemental Digital Content 5.** Outcomes reported in the included studies and the studies used for all meta-analyses.

| **Virus** | **Study** | **Outcomes reported in studies** | **Outcomes used in overall meta-estimates** | **Sensitivity analysis** | | | | **Subgroup analysis** | | |
| --- | --- | --- | --- | --- | --- | --- | --- | --- | --- | --- |
|  |  |  |  | **QA score ≥7** | **ICU admission only** | **Excl. mortality** | **Excl. suppl. oxygen** | **<2 y.o.** | **Developed countries** | **Developing countries** |
| **RSV** | Aikphaibul *et al.* (2021) | Combined: SO, MV, PHS (>7 days), M | combined |  |  |  |  |  |  | combined |
|  | Anderson *et al.* (2022) | Combined: SO, MV | combined | combined |  | combined |  | combined | combined |  |
|  | Cai *et al.* (2020) | Individual: ICU, MV, M | ICU; MV (for chronic lung disease only) | ICU; MV (for chronic lung disease only) | ICU | ICU; MV (for chronic lung disease only) | ICU; MV (for chronic lung disease only) |  | ICU; MV (for chronic lung disease only) |  |
|  | Chi *et al.* (2011) | Individual: ICU | ICU | ICU | ICU | ICU | ICU |  |  | ICU |
|  | Eski *et al.* (2021) | Individual: ICU | ICU | ICU | ICU | ICU | ICU |  |  | ICU |
|  | Ferolla *et al.* (2019) | Combined: SO, MV | Estimates from this study were not used in the meta-analysis | | | | | | | |
|  | Geoghegan *et al.* (2017) | Combined: RF (includes SO and MV) | combined | combined |  | combined |  | combined |  | combined |
|  | Greenberg *et al.* (2014) | Individual: ICU | ICU | ICU | ICU | ICU | ICU | ICU |  | ICU |
|  | Halasa *et al.* (2015) | Individual: SO, ICU, MV | ICU | ICU | ICU | ICU | ICU |  |  | ICU |
|  | Helfrich *et al.* (2015) | Individual: SO | SO | SO |  | SO |  | SO | SO |  |
|  | Hervas *et al.* (2012) | Individual: ICU, SO | ICU | ICU | ICU | ICU | ICU |  | ICU |  |
|  | Fischer Langley *et al.* (2013) | Combined: ICU, MV, M | combined | combined |  |  | combined |  |  | combined |
|  | Lu *et al.* (2015) | Individual: ICU | ICU |  | ICU | ICU | ICU | ICU |  | ICU |
|  | Meenaghan *et al.* (2020) | Individual: ICU | ICU |  | ICU | ICU | ICU |  | ICU |  |
|  | Moreno-Perez *et al.* (2014) | Individual: ICU, SO, MV | ICU | ICU | ICU | ICU | ICU |  | ICU |  |
|  | Moyes *et al.* (2013) | Individual: PHS (>5 days), M | PHS (>5 days) | PHS (>5 days) |  | PHS (>5 days) | PHS (>5 days) |  |  | PHS (>5 days) |
|  | Okubo *et al.* (2018) | Individual: ICU | ICU | ICU | ICU | ICU | ICU | ICU | ICU |  |
|  | Papenburg *et al.* (2012) | Combined: SO, ICU, PHS (>5 days) | combined | combined |  | combined |  |  | combined |  |
|  | Patel *et al.* (2019) | Combined: SO, MV, M | combined |  |  |  |  | combined |  | combined |
|  | Rodriguez *et al.* (2014) | Individual: ICU | ICU |  | ICU | ICU | ICU |  |  | ICU |
|  | Rodriguez-Martinez *et al.* (2022) | Individual: ICU, RF (includes SO, MV) | ICU | ICU | ICU | ICU | ICU |  |  | ICU |
|  | Sanchez-Luna *et al.* (2016) | Individual: M | M |  |  |  | M | M | M |  |
|  | Shmueli *et al.* (2021) | Combined: ICU, PHS (≥6 days) | combined | combined |  | combined | combined | combined |  | combined |
|  | Stagliano *et al.* (2015) | Combined: RS (includes SO, MV) | combined | combined |  | combined |  |  | combined |  |
|  | Van de Steen *et al.* (2016) | Individual: ICU | ICU |  | ICU | ICU | ICU | ICU | ICU |  |
|  | Viguria *et al.* (2018) | Individual: ICU | ICU | ICU | ICU | ICU | ICU |  | ICU |  |
|  | Zhang *et al.* (2014) | Combined: PHS (>5 days), MV | combined |  |  | combined | combined | combined |  | combined |
| **Influenza** | Cotes *et al.* (2012) | Individual: SO, ICU, MV, M | Estimates from this study were not used in the meta-analysis | | | | | | | |
|  | Kamidani *et al.* (2022) | Individual: ICU, MV, M | Estimates from this study were not used in the meta-analysis | | | | | | | |
| **SARS-CoV-2** | Martinez-Valdez *et al.* (2022) | M | Estimates from this study were not used in the meta-analysis | | | | | | | |

**Outcomes**: ICU= admission to intensive care unit, SO= supplementary oxygen, MV= mechanical ventilation, PHS= prolonged hospital stay, RF= respiratory failure, RS= respiratory support, M= mortality.
