# Supplementary figures and images for: Risk Factors for Poor Outcomes in Children Hospitalized With Virus-associated Acute Lower Respiratory Infections: A Systematic Review and Meta-analysis
Source: Pediatr Infect Dis J. 2024 Jan 26;43(5):467–76. doi: 10.1097/INF.0000000000004258 (PMC11003409; doi:10.1097/INF.0000000000004258)

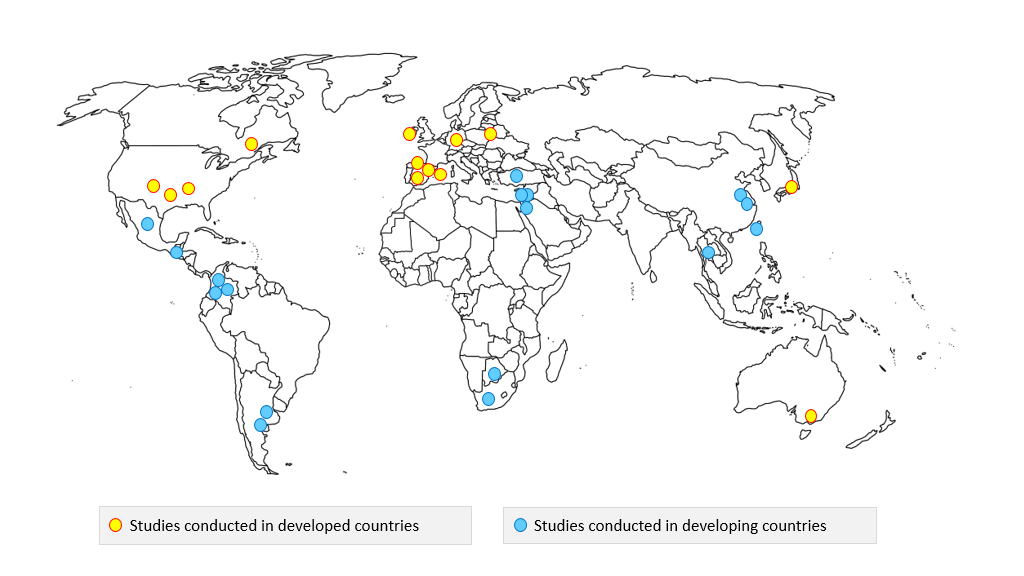
**Supplemental Digital Content 6.** Locations of the study sites included in the systematic review.

Supplement: Supplementary file 6 [file inf-43-0467-s006.docx]
