# Supplementary material for: Risk Factors for Poor Outcomes in Children Hospitalized With Virus-associated Acute Lower Respiratory Infections: A Systematic Review and Meta-analysis
Source: Pediatr Infect Dis J. 2024 Jan 26;43(5):467–76. doi: 10.1097/INF.0000000000004258 (PMC11003409; doi:10.1097/INF.0000000000004258)
Supplement: Supplementary file 7 [file inf-43-0467-s007.docx]

**Supplemental Digital Content 7.** Characteristics of all the studies included in the systematic review and meta-analysis.

| Study | Virus | Location | Region | Study period | Study design | Sample size | Age | Virus diagnostics | Case definition | Risk factors | Outcomes |
| --- | --- | --- | --- | --- | --- | --- | --- | --- | --- | --- | --- |
| Aikphaibul *et al.* (2021) | RSV | Bangkok, Thailand | Developing | Jan 2011 - Dec 2016 | Retrospective descriptive study | 361 | <5y | ICD-10, RDT, PCR | ALRI | CHD, CLD, GI, HEM, IC, P | SO, MV, PHS (>7 days), M |
| Anderson *et al.* (2022) | RSV | Melbourne, Australia | Developed | Jan 2017 - Dec 2019 | Retrospective cohort study | 970 | <2y | RT-PCR (NPA/NPS) | ALRI | CLD, CO, P, YA | SO, MV |
| Cai *et al.* (2020) | RSV | 84 hospitals in Germany | Developed | Jan 2009 - May 2018 | Retrospective cohort study | 8521 | <5y | ICD-10 | ARI | CHD, CLD, DS, GI, HEM, LBW, NEU, P, vitD, YA | ICU, MV, M |
| Chi *et al.* (2011) | RSV | Taiwan, China | Developing | 2004 - 2007 | Retrospective case-control study | 11081 | <5y | ICD-9 | B, P, ARI | CHD, CLD, HEM, MS, NEU, P, UD | ICU |
| Cotes *et al.* (2012) | Influenza | Bogota and Manizales, Colombia | Developing | April 2000 - Nov 2006 (flu seasons) | Retrospective study | 535 | <2y | ICD-10 | ARI | ACIP, non-ACIP | ICU, MV, SO, M |
| Eski *et al.* (2021) | RSV | Izmir, Turkey | Developing | Jan 2010 - Jan 2020 | Retrospective observational study | 906 | <5y | RT-PCR | ALRI | CO | ICU |
| Ferolla *et al.* (2019) | RSV | Buenos Aires, Argentina | Developing | 2012 - 2013 | Prospective study | 372 | <2y | DIFA (NPA) | ARI | AT, OC, SMO | SO, MV |
| Geoghegan *et al.* (2017) | RSV | Buenos Aires, Argentina | Developing | 2011 - 2013 | Prospective cross-sectional study | 2588 | <2y | DIFA, RT-PCR | ALRI | AT, nBF, CHD, DS, FAR, II, MS, NEU, OC, PH, SMO, UW, VM, YA | RF (includes SO and MV) |
| Greenberg *et al.* (2014) | RSV | Negev district, Israel | Developing | Nov 2004 - March 2011 (season Nov-March) | Prospective observational study | 1058 | <2y | n/a (NPW) | P | P | ICU |
| Halasa *et al.* (2015) | RSV | Amman, Jordan | Developing | March 2010 - March 2013 | Prospective cohort study | 1381 | <2y | RT-PCR (NPA/NPS) | ARI | nBF, MS, SMO, UD, vitD | SO, ICU, MV |
| Helfrich *et al.* (2015) | RSV | USA | Developed | Oct 2005 - April 2011 | Retrospective cohort study | 7597 | <2y | ICD-9 | ARI, B, P | P | SO |
| Hervas *et al.* (2012) | RSV | Mallorca, Spain | Developed | Jan 1995 - Dec 2006 | Retrospective study | 1495 | ≤2y | ELI, VC (NPA/NPW) | B | CHD, P | ICU |
| Kamidani *et al.* (2022) | Influenza | USA | Developed | 2010-2019 (season Oct-April) | Prospective study | 7860 | <5y | RDT, PCR, VC, IF | IAH | YA | ICU, MV, M |
| Fischer Langley *et al.* (2013) | RSV | 3 study sites in Guatemala | Developing | Nov 2007 - July 2010 | Prospective study | 549 | <5y | RT-PCR (NPS) | ARI | CHD, CLD, CO, MS, P, UD, YA | ICU, MV, M |
| Lu *et al.* (2015) | RSV | Suzhou, China | Developing | Jan 2010 - Dec 2014 | Retrospective study | 374 | ≤28d | DIFA (NPA) | ALRI | CO, LBW, MS, P, UD | ICU |
| Martinez-Valdez *et al.* (2022) | SARS-CoV-2 | Mexico | Developing | March 2020 - Sep 2021 | Retrospective cross-sectional study | 2148 | <1y | PCR, RDT | B | CHD, CKD, IC, UD | M |
| Meenaghan *et al.* (2020) | RSV | Dublin, Ireland | Developed | Nov 2014 - Feb 2017 (season Nov - Feb) | Retrospective case-control study | 557 | ≤2y | n/a (NPA) | B | MS, OW, P, UD, UW, YA | ICU |
| Moreno-Perez *et al.* (2014) | RSV | 26 hospitals in Spain | Developed | Dec 2011 - March 2012 | Prospective observational study | 685 | <5y | ELISA (NPA) | ARI | CHD, CLD, IC, MET, NEU, UD | ICU, SO, MV |
| Moyes *et al.* (2013) | RSV | 4 surveillance sites in South Africa | Developing | Jan 2010 - Dec 2011 | Prospective observational study | 1157 | <5y | RT-PCR (NPA) | ALRI | CO, IC, MS, P, YA | PHS (>5d), M |
| Okubo *et al. (*2018) | RSV | Tokyo, Japan | Developed | July 2010 - March 2015 | Retrospective study | 42698 | <1y | ICD-10 | Br, P | OW, UW | ICU |
| Papenburg *et al.* (2012) | RSV | Quebec, Canada | Developed | 2006 - 2010 (season Nov - April) | Prospective cohort study | 467 | <3y | RT-PCR (NPA) | ARI | CO, P, YA | ICU, SO, PHS (>5d) |
| Patel *et al.* (2019) | RSV | Gaborone, Botswana | Developing | April 2012 - June 2016 | Prospective cohort study | 123 | 1-23m | PCR (NPS) | ALRI | nBF, CO, IC, MS, PH, UW, YA | SO, MV, M |
| Rodriguez *et al.* (2014) | RSV | Bogota, Colombia | Developing | May 2009 - April 2011 | Retrospective cohort study | 2147 | <3y | RDT (NPA) | ALRI | CHD, CKD, CLD, CO, IC, P, YA | ICU |
| Rodriguez-Martinez *et al.* (2022) | RSV | Colombia | Developing | Jan 2018 - Dec 2018 | Retrospective cross-sectional study | 1215 | <5y | IIFA, RT-PCR | ALRI | CHD, II, IND, LES, MS, UW, YA | ICU, RF (includes SO, MV) |
| Sanchez-Luna *et al.* (2016) | RSV | Spain | Developed | 2004 - 2012 | Retrospective study | 63990 | <1y | ICD-9 | B | CHD, CLD, DS, NEU, P | M |
| Shmueli *et al.* (2021) | RSV | Petach Tikva, Israel | Developing | 2014 - 2018 (season Oct-April) | Retrospective study | 1124 | <2y | PCR | B | CHD, CLD, CO, DS, IC, MS, NEU, P, UD, YA | ICU, PHS (≥6d) |
| Stagliano *et al.* (2015) | RSV | USA | Developed | Oct 2005 - April 2011 | Retrospective cohort study | 9048 | <3y | ICD-9 | ALRI | DS | RS (includes SO, MV) |
| Van de Steen *et al.* (2016) | RSV | 12 countries in Central and Eastern Europe | Developed | 2009 - 2011 (season Oct - April) | Retrospective cohort study | 1423 | <1y | RDT | ALRI | P | ICU |
| Viguria *et al*. (2018) | RSV | Northern Spain | Developed | 2010 - 2015 (RSV seasons) | Retrospective study | 647 | <5y | ICD-9, RDT, RT-PCR | B, P | MS, UD, YA | ICU |
| Zhang *et al.* (2014) | RSV | Shanghai, China | Developing | March 2011 - Feb 2012 | Retrospective study | 913 | <1y | DIFA (S/ETA) | ALRI | AT, CHD, CLD, IC, LBW, LES, MS, OC, P, SMO, UD, YA | PHS (>5d), MV |

**Virus diagnostics:** RDT=rapid diagnostic test, PCR= polymerase chain reaction, RT-PCR= reverse transcriptase polymerase chain reaction, VC= viral culture, ELI= enzyme-linked immunoassay, ELISA= enzyme-linked immunosorbent assay, IFA= immunofluorescence, DIFA= direct immunofluorescence assay, IIFA= indirect immunofluorescence assay, ICD-9= International Classification of Diseases, 9th Revision, ICD-10= International Classification of Diseases, 10th Revision, NPA= nasopharyngeal aspirate, NPS= nasopharyngeal swab, NPW= nasopharyngeal wash, ETA= endotracheal aspirate, S= sputum.

**Case definitions:** ALRI= acute lower respiratory infection, ARI= acute respiratory infection, IAH= influenza-associated hospitalisation, B= bronchiolitis, Br= bronchitis, P= pneumonia.

**Risk factors:** ACIP= Advisory Committee for Immunization Practices (ACIP) - identified clinical conditions that increase the risk for acquiring influenza complications (chronic cardiovascular disease, chronic lung disease, asthma without other chronic lung diseases, metabolic or endocrine disorders, immunosuppression), non-ACIP= clinical conditions not identified by Advisory Committee for Immunization Practices (ACIP) as increasing the risk for acquiring influenza complications (nutritional disorders, CNS disorders, congenital malformations, digestive disorders, prematurity), AT = family history of atopy, nBF = lack of breastfeeding, CHD = congenital heart disease, CKD=chronic kidney disease, CLD = chronic lung disease, CO = viral coinfection, DS = Down syndrome, FAR= living far from hospital, GI = gastrointestinal diseases, HEM = hematologic conditions, IC = immunocompromised status, II= incomplete immunisation for age, IND= indigenous ethnicity, LBW= low birth weight, LES = low socioeconomic status, MET = metabolic conditions, MS = male sex, NEU= neurologic disease, OC = over-crowding, OW= overweight, P = prematurity, PH= precarious home, SMO = smoke exposure, UD = underlying diseases, UW = underweight, vitD= vitamin D deficiency, VM= vulnerable mother, YA = young age.

**Outcomes**: ICU= admission to intensive care unit, SO= supplementary oxygen, MV= mechanical ventilation, PHS= prolonged hospital stay, RF= respiratory failure, RS= respiratory support, M= mortality.
